# Quantifying association and disparities between diabetes complications and COVID-19 outcomes: A retrospective study using electronic health records

**DOI:** 10.1371/journal.pone.0286815

**Published:** 2023-09-28

**Authors:** Ni Luh Putu S. P. Paramita, Joseph K. Agor, Maria E. Mayorga, Julie S. Ivy, Kristen E. Miller, Osman Y. Ozaltin

**Affiliations:** 1 Operations Research Graduate Program, North Carolina State University, Raleigh, North Carolina, United States of America; 2 School of Mechanical, Industrial, and Manufacturing Engineering, Oregon State University, Corvallis, Oregon, United States of America; 3 Edward P. Fitts Department of Industrial and Systems Engineering, Raleigh, North Carolina, United States of America; 4 National Center for Human Factor in Healthcare, MedStar Health, Washington, District of Columbia, United States of America; National Cancer Institute of Brazil (INCA/MS), BRAZIL

## Abstract

**Background:**

Despite established relationships between diabetic status and an increased risk for COVID-19 severe outcomes, there is a limited number of studies examining the relationships between diabetes complications and COVID-19-related risks. We use the Adapted Diabetes Complications Severity Index to define seven diabetes complications. We aim to understand the risk for COVID-19 infection, hospitalization, mortality, and longer length of stay of diabetes patients with complications.

**Methods:**

We perform a retrospective case-control study using Electronic Health Records (EHRs) to measure differences in the risks for COVID-19 severe outcomes amongst those with diabetes complications. Using multiple logistic regression, we calculate adjusted odds ratios (OR) for COVID-19 infection, hospitalization, and in-hospital mortality of the case group (patients with diabetes complications) compared to a control group (patients without diabetes). We also calculate adjusted mean difference in length of stay between the case and control groups using multiple linear regression.

**Results:**

Adjusting demographics and comorbidities, diabetes patients with renal complications have the highest odds for COVID-19 infection (OR = 1.85, 95% CI = [1.71, 1.99]) while those with metabolic complications have the highest odds for COVID-19 hospitalization (OR = 5.58, 95% CI = [3.54, 8.77]) and in-hospital mortality (OR = 2.41, 95% CI = [1.35, 4.31]*)*. The adjusted mean difference (MD) of hospital length-of-stay for diabetes patients, especially those with cardiovascular (MD = 0.94, 95% CI = [0.17, 1.71]) or peripheral vascular (MD = 1.72, 95% CI = [0.84, 2.60]) complications, is significantly higher than non-diabetes patients. African American patients have higher odds for COVID-19 infection (OR = 1.79, 95% CI = [1.66, 1.92]) and hospitalization (OR = 1.62, 95% CI = [1.39, 1.90]) than White patients in the general diabetes population. However, White diabetes patients have higher odds for COVID-19 in-hospital mortality. Hispanic patients have higher odds for COVID-19 infection (OR = 2.86, 95% CI = [2.42, 3.38]) and shorter mean length of hospital stay than non-Hispanic patients in the general diabetes population. Although there is no significant difference in the odds for COVID-19 hospitalization and in-hospital mortality between Hispanic and non-Hispanic patients in the general diabetes population, Hispanic patients have higher odds for COVID-19 hospitalization (OR = 1.83, 95% CI = [1.16, 2.89]) and in-hospital mortality (OR = 3.69, 95% CI = [1.18, 11.50]) in the diabetes population with no complications.

**Conclusions:**

The presence of diabetes complications increases the risks of COVID-19 infection, hospitalization, and worse health outcomes with respect to in-hospital mortality and longer hospital length of stay. We show the presence of health disparities in COVID-19 outcomes across demographic groups in our diabetes population. One such disparity is that African American and Hispanic diabetes patients have higher odds of COVID-19 infection than White and Non-Hispanic diabetes patients, respectively. Furthermore, Hispanic patients might have less access to the hospital care compared to non-Hispanic patients when longer hospitalizations are needed due to their diabetes complications. Finally, diabetes complications, which are generally associated with worse COVID-19 outcomes, might be predominantly determining the COVID-19 severity in those infected patients resulting in less demographic differences in COVID-19 hospitalization and in-hospital mortality.

## Introduction

Coronavirus Disease 2019 (COVID-19) is an infectious disease caused by the Severe Acute Respiratory Syndrome Coronavirus 2 (SARS-CoV-2) virus. On March 20, 2020, following a total of 234,073 confirmed cases and 9,840 deaths globally [1], the World Health Organization (WHO) declared COVID-19 a pandemic. Demographic factors such as age, sex, race, and ethnicity are associated with the risk for COVID-19 infection, hospitalization, and death [2]. Furthermore, individuals with underlying health conditions have a higher risk for severe COVID-19 outcomes [3, 4]. The most common comorbidities among patients hospitalized with COVID-19 include hypertension, obesity and diabetes [5, 6]. In this study, we analyze the association between diabetes complications and COVID-19 outcomes. We are specifically interested in COVID-19 infection, hospitalization, hospital length of stay, and in-hospital mortality. Type 1 and Type 2 diabetes are associated with increased chance of hospitalization for COVID-19 and in-hospital mortality [7–10]. Cardiovascular conditions and hospitalizations, especially those related to heart failure, stroke, and myocardial infarction, are associated with increased COVID-19 related mortality in patients with diabetes [10]. Lim et al. [11] proposed several pathophysiological mechanisms leading to increased cardiovascular mortality after infection with SARS-CoV-2 in patients with diabetes such as increased levels of inflammatory mediators in the blood (e.g., lipopolysaccharide, cytokines, and metabolites), insulin resistance, and vascular endothelial damage. Zhou et al. [12] provided a synopsis of the reasons for increased severity of COVID-19 in patients with diabetes including aggravated inflammatory storm, immune system dysfunction, and diabetes-related comorbidities such as lung injury and renal damage. Guo et al. [13] investigated the pathophysiological mechanisms behind the deterioration of COVID-19 patients with diabetes, and found that they had higher ferritin and coagulation index compared to patients without diabetes, suggesting that COVID-19 patients with diabetes are more susceptible to an inflammatory storm. Furthermore, they observed higher odds of mortality in patients with diabetic complications. However, not all complications were represented in their study cohort. Additionally, having a history of microvascular diabetic complications (e.g., diabetic kidney disease, severe diabetic retinopathy, and history of diabetic foot ulcer) was associated with a reduced chance of discharge within 28 days of hospital admission for those infected with COVID-19 [14].The focus of this study was to quantify this association amongst those hospitalized and did not investigate the association of these complications with being hospitalized with COVID-19.

Diabetes may cause macrovascular and microvascular complications based on the injurious effects of hyperglycemia [15]. Hyperglycemia (high blood glucose) is considered a metabolic complication of diabetes [16, 17] and there are many studies that establish a relationship between hyperglycemia and severe COVID-19 outcomes, especially in patients with diabetes [18–23]. These studies focus on hyperglycemia alone and, despite being a common metabolic complication amongst diabetes patients, do not investigate a more general relationship between diabetes related metabolic complications and COVID-19 outcomes. Similar analysis has been done on the relationship between COVID-19 and other specific metabolic complications among the diabetes population including ketoacidosis [24–28] and hypoglycemia [29]. Associations between specific diabetic complications and COVID-19 extends to macrovascular complications including coronary artery disease [30], peripheral arterial disease [31], and stroke [32] as well as other microvascular complications such as diabetic nephropathy [33, 34], neuropathy [35], and retinopathy [34, 36].

Although there is an abundance of work investigating the relations between diabetes and COVID-19, previous research has focused on either the general diabetes population (i.e., not focused on complications) or patients with specific diabetic complications (e.g., hyperglycemia as a metabolic complication or peripheral arterial disease as a cardiovascular complication). There are currently no studies that compare the association of different diabetic complications with COVID-19 outcomes. Furthermore, there are no studies investigating demographic disparities in COVID-19 outcomes among patients with diabetic complications.

One of the objectives of this work is to understand the association of different microvascular (i.e., renal, neurological, ocular) and macrovascular (i.e., cardiovascular, peripheral vascular, cerebrovascular, metabolic) diabetic complications to COVID-19 outcomes. We specifically consider COVID-19 infection, hospitalization, in-hospital mortality and hospital length of stay outcomes. We also aim to investigate demographic disparities in COVID-19 outcomes among patients with diabetic complications. We accomplish these objectives through a retrospective cohort study analyzing the observations of the considered COVID-19 outcomes in patients with diabetic complications across seven different body systems. Our findings provide clinicians with actionable information when caring for patients that have diabetic complications by indicating which complications, if any, have a higher association with adverse COVID-19 outcomes.

## Materials and methods

### Data

This is a retrospective study that uses personal protected health information derived from electronic medical records at MedStar Health, a healthcare system that operates 10 hospitals in the Baltimore-Washington metropolitan area. The data was fully anonymized before being accessed by the research team. The study was approved by North Carolina State University’s Institutional Review Board (IRB No. 21006). The period of observation is between January 2019 to December 2020 including 1,389,115 unique patients from 6,265,821 encounters with patient level information. We consider 2019 only to identify the diabetic status of patients based on their medical history before the pandemic. Furthermore, by considering the first year of the COVID-19 pandemic when disease mitigation measures (e.g, antiviral drugs and vaccines) were not available and treatment strategies were not well understood, we focus on a period when diabetes patients were at a higher risk for adverse COVID-19 outcomes.

From this initial patient population, we exclude 6,008 patients who died in 2019. There are 156 patients (0.011%) with missing birthdate, 639 patients (0.046%) with missing sex, 169,276 patients (12.19%) with missing race, and 191,555 patients (13.79%) with missing ethnicity information. We exclude patients with missing birthdate and retain other patients with missing information in the analysis because excluding them all would significantly reduce the population size. We do not impute missing values for sex, race and ethnicity but instead create a missing category. After this exclusion, the final population includes 1,382,951 patients.

We split the population into a case group with 142,807 patients (10.33% of the patient population) and a control group with the rest of the patients. The patients in the case group are those who had at least one diabetes-related encounter during or before 2019 as indicated in their past medical history. A diabetes-related encounter is defined as an encounter with at least one of the ICD-10 codes associated with diabetes (E10—E14). Finally, our data includes 43,473 COVID-19-related encounters from 37,245 patients (2.69% of the patient population) with a positive COVID-19 test, and 8,431 of these encounters resulted in hospitalization of 6,988 patients throughout the observation period.

#### Observation generation

The outcomes of interest in this work are COVID-19 infection, COVID-19 hospitalization, COVID-19 length of stay, and COVID-19 in-hospital mortality. A patient is said to have a COVID-19 infection if that patient had at least one COVID-19-related encounter during the observation period. If at least one of these COVID-19-related encounters is classified as an inpatient encounter, we say that the patient had a COVID-19 hospitalization. Thus, COVID-19 is not necessarily the primary or only cause for this hospitalization. For patients with a COVID-19 hospitalization, the COVID-19 length of stay is the duration of the COVID-19 hospitalization encounter or the maximum duration if the patient had more than one COVID-19-related hospitalization. COVID-19 in-hospital mortality is identified through the discharge disposition of the last COVID-19 hospitalization.

**Fig 1:**
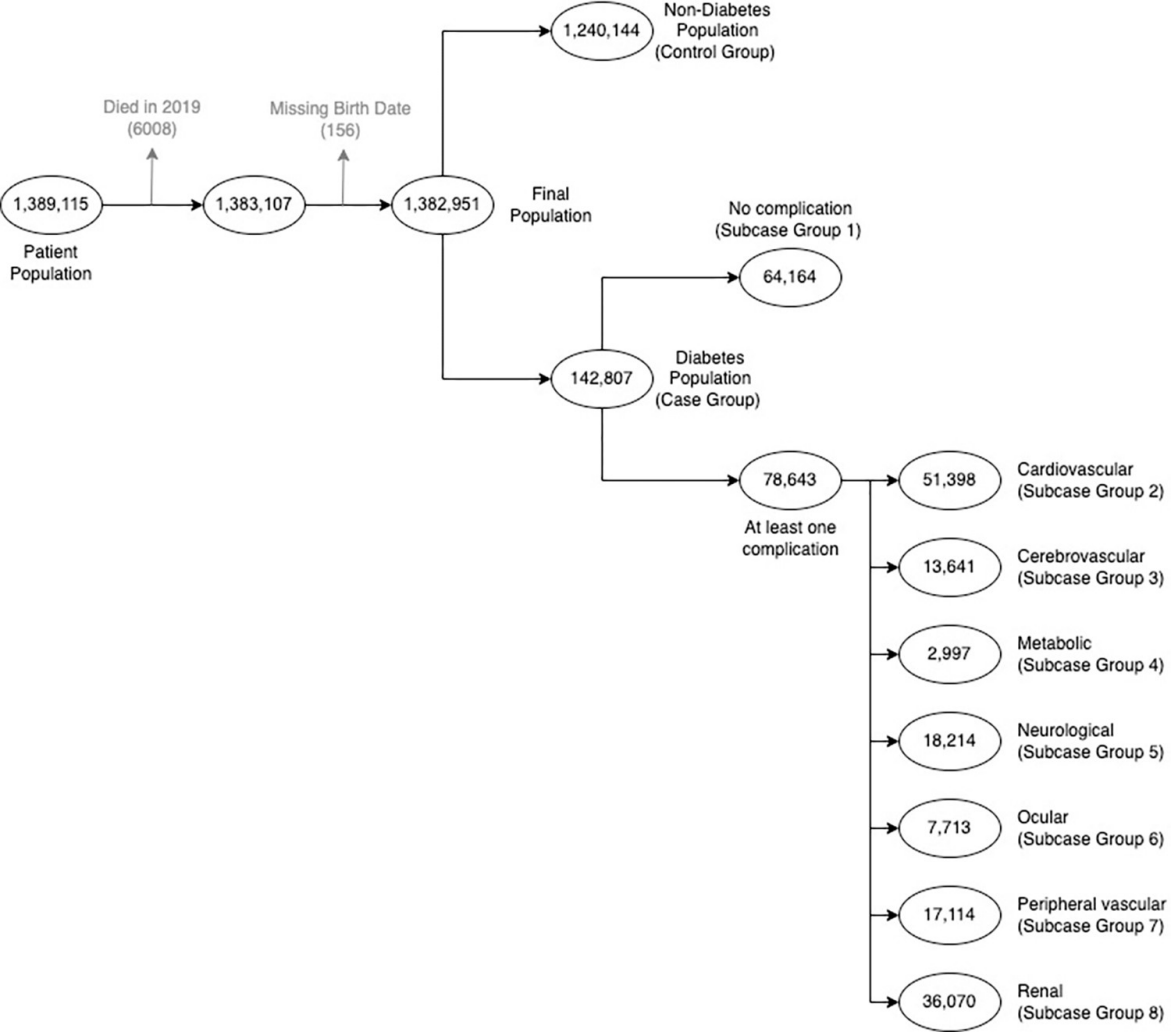
Case and control groups based on diabetes complication indicators.

The covariates of interest are diabetes status and its complications. Diabetes complications are defined based on the adapted Diabetes Complications Severity Index (aDCSI) [16]. The aDCSI classifies diabetes complications into seven categories: cardiovascular, renal, ocular, peripheral vascular, cerebrovascular, neurological and metabolic complications. We use the translation of aDCSI to ICD-10 [17] to assign patients in the case group to the eight subcase groups, that is, a group of diabetes patients without any complications and seven groups of diabetes patients with different complications. For example, if a patient had at least one encounter with ICD-10 codes associated with cardiovascular complication as defined in aDCSI, then we include that patient in the subcase group of diabetes patients with cardiovascular complication. Note that a patient may be included in multiple complication groups. Fig 1 outlines the process of generating the case and control groups based on diabetes status and its complications.

**Fig 2:**
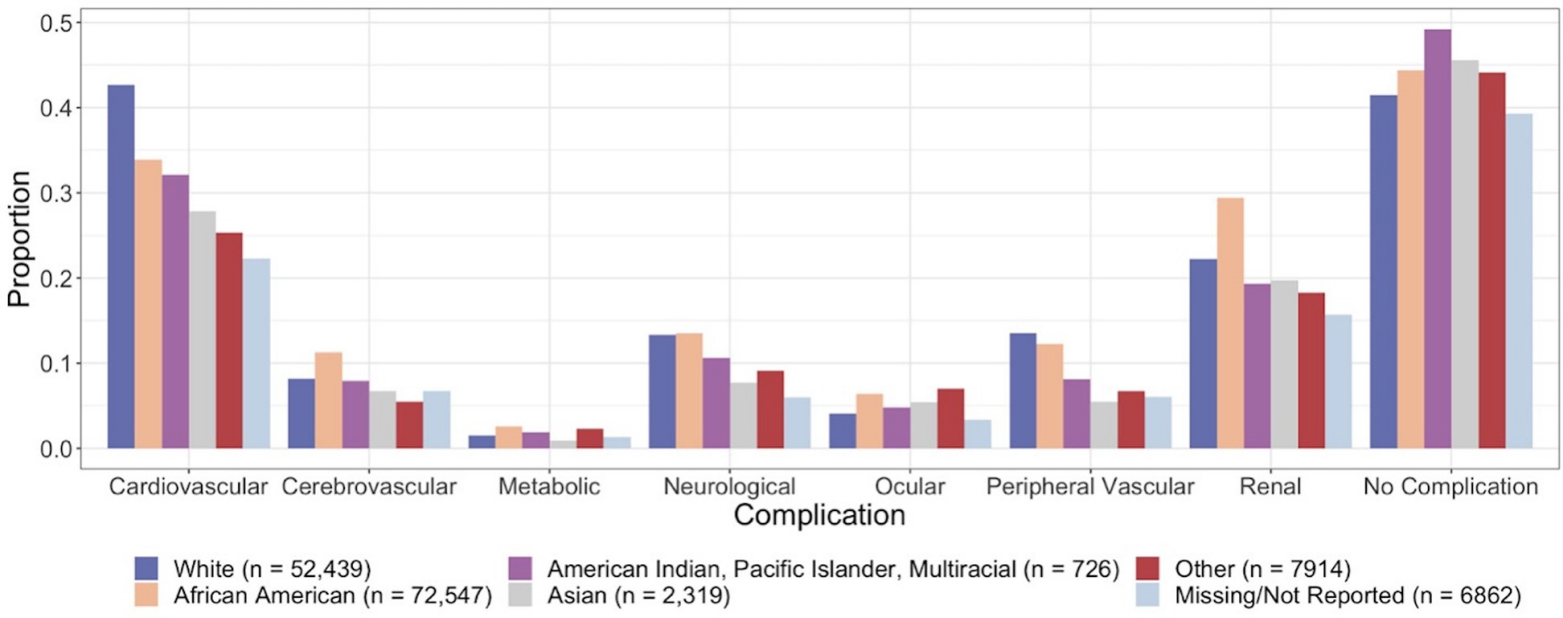
Proportion of diabetes patients by complications among each race.

**Fig 3:**
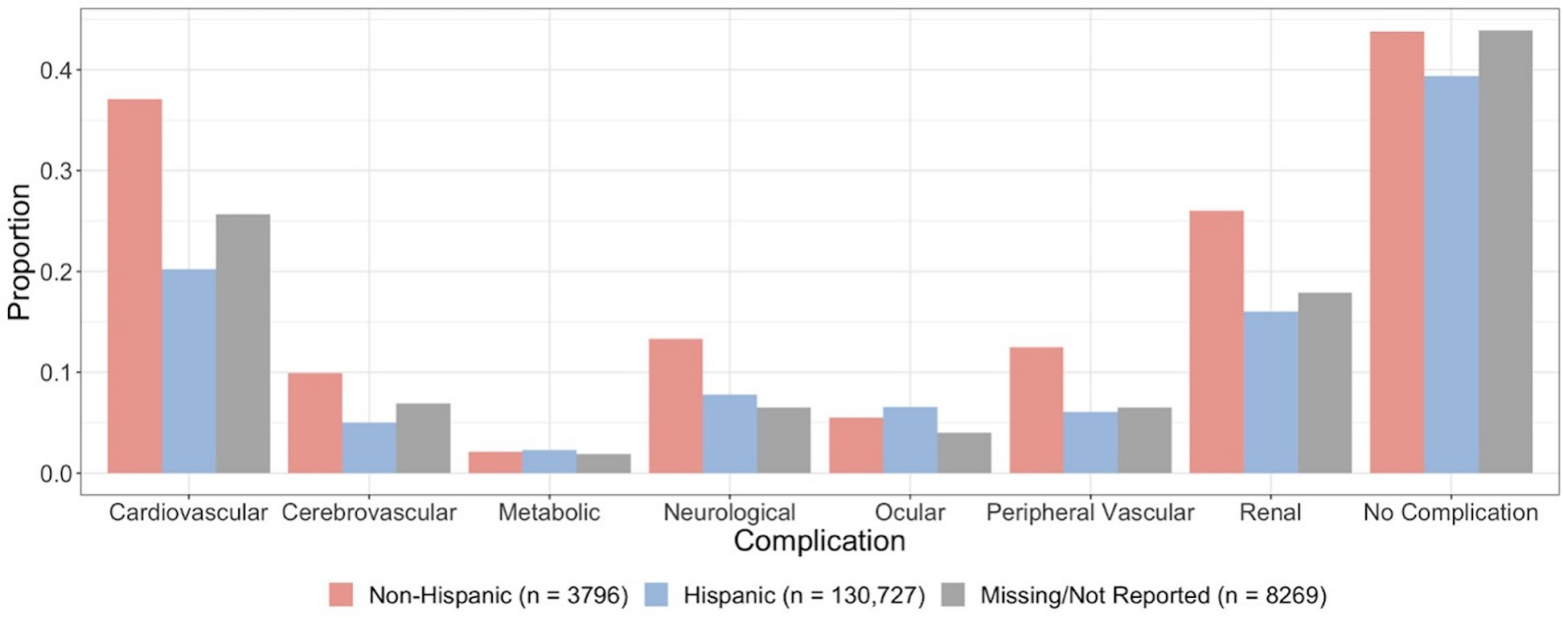
Proportion of diabetes patients by complications among each ethnicity.

We consider age, sex, race, and ethnicity as confounding factors in our analysis. Figs 2 and 3 present the proportion of diabetes patients by complication among each race and ethnicity, respectively. We also consider a list of suspected underlying medical conditions associated with more severe COVID-19 illness, other than diabetes, as confounders related to the outcomes and covariates of interest. This includes obesity, hypertension, chronic kidney disease, chronic lower respiratory diseases, substance use disorder, heart disease, cancer, chronic liver disease, stroke, dementia, mood disorders, schizophrenia, rheumatoid arthritis, lupus, psoriasis, and disorders involving an immune mechanism [3, 4]. A patient is identified as having an underlying medical condition if they had at least one encounter during or before 2019 with ICD-10 codes associated with those conditions.

### Statistical analysis

We calculate the adjusted odds ratio for COVID-19 infection, hospitalization, and in-hospital mortality of the case group compared to the control group. The odds ratio is an appropriate measure of risk in case-control studies under the “uncommon disease” assumption, i.e. when the probability of an outcome is less than 0.1 or 0.2 [37, 38]. We also calculate the adjusted mean difference in length of stay for COVID-19 hospitalization between diabetes and non-diabetes patients. From a clinical perspective, length of stay has been considered as a meaningful outcome and a potential indicator for the quality of patient care [39, 40].

Several factors, including a patient’s demographic characteristics and underlying medical conditions, influence the relationship between diabetic status and COVID-19 outcomes of interest. We use multiple logistic regression to calculate adjusted odds ratios (AOR) to consider the influence of confounding factors [41]. The multiple logistic regression model is formulated as:

logit(P(Y=1))=α+βx+γz+ϵ

where *Y* is the outcome of interest, *x* is the diabetic status, *z* is a vector of demographics and underlying medical conditions, *ϵ* is the error term. Then the estimated AOR that associates diabetic status to the outcome of interest (e.g. COVID-19 infection) is *exp* (*β*).

To compute the adjusted mean difference (AMD) for length of stay, we use multiple linear regression [42]. The multiple linear regression model is formulated as follows:

Y=α+βx+γz+ϵ

where *Y* is the COVID-19 hospital length of stay. The estimated difference in average length of stay between case and control groups adjusted for demographics and underlying medical conditions is given by *β*.

## Results

We conduct a separate analysis for all patients with diabetes (case group), diabetes patients without complications (subcase group 1), and diabetes patients with complications (subcase groups 2–7). For each analysis, the control group includes patients without diabetes. We present the AOR for COVID-19 infection, hospitalization, in-hospital mortality and AMD of hospital length of stay along with the p-values. Table 1 presents the demographic characteristics of the study population. We perform Pearson’s Chi-Square test to identify differences between the demographic characteristics of the case and control groups that should be adjusted in the analysis. We observe statistically significant differences (p < 0.001) in Sex, Age, Race, and Ethnicity.

**Table 1:** Patients with diabetes and COVID-19 infection by patient subgroup.

Patient subgroup	Study population	COVID-19	Diabetes	COVID-19 and Diabetes
Total	1,382,951	37,245	142,807	4,690
Sex (% of total)				
Female	793,373 (57.37%)	19,938 (53.53%)	77,088 (53.98%)	2,505 (53.41%)
Male	588,939 (42.59%)	17,280 (46.40%)	65,702 (46.01%)	2,184 (46.57%)
Missing/not reported	639 (0.04%)	27 (0.07%)	17 (0.01%)	1 (0.02%)
Age (% of total)				
< 18	142,865 (10.33%)	1,844 (4.95%)	366 (0.26%)	1 (0.02%)
18–39	437,046 (31.60%)	15,311 (41.11%)	8,503 (5.95%)	343 (7.31%)
40–64	480,076 (34.71%)	13,793 (37.03%)	58,511 (40.97%)	2,218 (47.29%)
≥ 65	322,964 (23.36%)	6,297 (16.91%)	75,427 (52.82%)	2,128 (45.38%)
Race (% of total)				
African American	457,387 (33.07%)	11,690 (31.39%)	72,547 (50.80%)	2,780 (59.28%)
Asian	26,196 (1.89%)	359 (0.96%)	2,319 (1.62%)	42 (0.90%)
White	625,878 (45.26%)	12,373 (33.22%)	52,439 (36.72%)	1,100 (23.45%)
Other	91,831 (6.64%)	3,754 (10.08%)	7,914 (5.54%)	490 (10.45%)
American Indian, Pacific Islander, Multiracial	12,383 (0.89%)	255 (0.68%)	726 (0.51%)	22 (0.47%)
Missing/not reported	169,276 (12.25%)	8,814 (23.67%)	6,862 (4.81%)	256 (5.45%)
Ethnicity (% of total, 0.03% “Multiple” ethnicity is not reported)				
Hispanic	51,258 (3.71%)	2,605 (6.99%)	3,796 (2.66%)	305 (6.50%)
Non-Hispanic	1,139,679 (82.40%)	26,262 (70.51%)	130,727 (91.54%)	3,987 (85.01%)
Missing/not reported	191,555 (13.86%)	8,315 (22.33%)	8,269 (5.79%)	395 (8.42%)

### COVID-19 infection and hospitalization

Table 2 reports the adjusted odds ratios of diabetes patients for COVID-19 infection and hospitalization. The odds ratios of diabetes patients for COVID-19 infection is significant and greater than one regardless of the complication type. The strongest effect of diabetic status on COVID-19 infection is observed in patients with renal complications (OR = 1.85, 95% CI = [1.71, 1.99]) followed by those with neurological complications (OR = 1.82, 95% CI = [1.67, 1.98]). The odds ratios of diabetes patients with cardiovascular, cerebrovascular and ocular complications for COVID-19 hospitalization is greater than one but they are not statistically significant. The strongest effect of diabetic status on COVID-19 hospitalization is observed in patients with metabolic complications (OR = 5.58, 95% CI = [3.54, 8.77]) followed by those with peripheral vascular complications (OR = 1.82, 95% CI = [1.46, 2.26]).

**Table 2:** Adjusted odds ratios for COVID-19 infection and hospitalization (95% CI) [p-value].

	COVID-19 Infection	COVID-19 Hospitalization
**Diabetes**	1.43 (1.38, 1.49) [p < 0.001]^†^	1.53 (1.41, 1.67) [p < 0.001]^†^
**Cardiovascular complication**	1.56 (1.46, 1.66) [p < 0.001]^†^	1.07 (0.92, 1.25) [p = 0.361]
**Cerebrovascular complication**	1.74 (1.55, 1.94) [p < 0.001]^†^	1.09 (0.84, 1.41) [p = 0.538]
**Metabolic complication**	1.75 (1.46, 2.10) [p < 0.001]^†^	5.58 (3.54, 8.77) [p < 0.001]^†^
**Neurological complication**	1.82 (1.67, 1.98) [p < 0.001]^†^	1.62 (1.33, 1.99) [p < 0.001]^†^
**Ocular complication**	1.80 (1.60, 2.03) [p < 0.001]^†^	1.24 (0.93, 1.66) [p = 0.137]
**Peripheral vascular complication**	1.77 (1.62, 1.94) [p < 0.001]^†^	1.82 (1.46, 2.26) [p < 0.001]^†^
**Renal complication**	1.85 (1.71, 1.99) [p < 0.001]^†^	1.80 (1.52, 2.14) [p < 0.001]^†^
**No complication**	1.39 (1.32, 1.46) [p < 0.001]^†^	1.47 (1.32, 1.65) [p < 0.001]^†^

^†^significant at *α* = 0.05

### COVID-19 hospitalization outcomes

Table 3 presents the adjusted mean difference in length of stay and the adjusted odds ratios for in-hospital mortality. The average hospital length of stay for diabetes patients, especially those with cardiovascular or peripheral vascular complications is significantly higher than non-diabetes patients. In general, the odds of COVID-19 in-hospital mortality for diabetes patients are higher than non-diabetes patients. The odds ratios are statistically significant for the case groups of diabetes patients with metabolic, neurological, or renal complications. The highest significant odds ratio of in-hospital mortality is observed for diabetes patients with metabolic complications (OR = 2.41, 95% CI = [1.35, 4.31]).

**Table 3:** Adjusted mean difference of COVID-19 hospital length of stay and odds ratio of COVID-19 in-hospital mortality (95% CI) [p-value].

	Hospital Length of Stay	In-Hospital Mortality
**Diabetes**	0.76 (0.31, 1.22) [p = 0.001]^†^	1.18 (1.02, 1.38) [p = 0.031]^†^
**Cardiovascular complication**	0.94 (0.17, 1.71) [p = 0.016]^†^	1.12 (0.91, 1.38) [p = 0.293]
**Cerebrovascular complication**	0.24 (-1.56, 2.04) [p = 0.795]	1.26 (0.91, 1.74) [p = 0.170]
**Metabolic complication**	1.41 (-0.24, 3.05) [p = 0.093]	2.41 (1.35, 4.31) [p = 0.003]^†^
**Neurological complication**	-0.08 (-1.81, 1.64) [p = 0.924]	1.49 (1.12, 1.97) [p = 0.006]^†^
**Ocular complication**	-1.23 (-5.25, 2.78) [p = 0.547]	1.27 (0.86, 1.87) [p = 0.224]
**Peripheral vascular complication**	1.72 (0.84, 2.60) [p < 0.001]^†^	1.20 (0.92, 1.57) [p = 0.178]
**Renal complication**	1.15 (0, 2.31) [p = 0.050]	1.41 (1.14, 1.75) [p = 0.002]
**No complication**	0.43 (-0.22, 1.08) [p = 0.196]	1.14 (0.88, 1.49) [p = 0.318]

^†^significant at *α* = 0.05

### Demographic disparities in COVID-19 outcomes

In this section we present the results of a comparison between diabetes patients with and without complications in different demographic groups. Table 4 presents the adjusted odds ratios for COVID-19 infection, hospitalization, in-hospital mortality and the mean differences of hospital length of stay between different demographic groups. This analysis is separately performed for all diabetes patients, diabetes patients with complications, and diabetes patients with no complications. We perform the disparity analysis between the case and control groups of a demographic factor by adjusting for other demographic factors and comorbidities. For example, to calculate the AOR for COVID-19 in-hospital mortality between seniors (65+) and younger adults (18–39), we adjust for sex, race, ethnicity and comorbidities.

**Table 4:** Odds ratios of COVID-19 infection, hospitalization, in-hospital mortality and mean difference of COVID-19 hospital length of stay in diabetes patients (95% CI) [p-value].

	All Diabetes Patients				Diabetes Patients with Complication				Diabetes Patients with No Complication			
Case/ Control	Infection	*Hosp.	Hospital Length of Stay	In-Hospital Mortality	Infection	Hosp.	Hospital Length of Stay	In-Hospital Mortality	Infection	Hosp.	Hospital Length of Stay	In-Hospital Mortality
Female/ Male	0.91 (0.85, 0.96) [p = 0.001]^†^	0.73 (0.64, 0.83) [p < 0.001]^†^	-0.63 (-1.37, 0.11) [p = 0.093]	0.75 (0.60, 0.93) [p = 0.010]^†^	0.95 (0.88, 1.03) [p = 0.227]	0.90 (0.75, 1.07) [p = 0.234]	-0.58 (-1.49, 0.35) [p = 0.226]	0.73 (0.57, 0.93) [p = 0.012]^†^	0.85 (0.77, 0.93) [p < 0.001]^†^	0.59 (0.48, 0.72) [p < 0.001]^†^	-0.61 (-1.87, 0.64) [p = 0.340]	0.84 (0.51, 1.38) [p = 0.491]
Senior (65+) / Younger adult (18–39)	0.64 (0.55, 0.73) [p < 0.001]^†^	2.54 (1.89, 3.42) [p < 0.001]^†^	1.79 (0.17, 3.41) [p = 0.030]^†^	4.26 (1.79, 10.12) [p = 0.001]^†^	0.62 (0.49, 078) [p < 0.001]^†^	1.73 (1.08, 2.78) [p = 0.022]^†^	1.80 (-0.50, 4.10) [p = 0.126]	2.43 (0.93, 6.38) [p = 0.071]	0.63 (0.52, 0.75) [p < 0.001]^†^	3.13 (2.05, 4.78) [p < 0.001]^†^	1.73 (-0.68, 4.14) [p = 0.159]	15.01 (1.89, 119.21) [p = 0.010]^†^
Senior (65+) / Older adult (40–64)	0.70 (0.66, 0.75) [p < 0.001]^†^	1.89 (1.64, 2.18) [p < 0.001]^†^	0.03 (-0.79, 0.86) [p = 0.941]	2.15 (1.66, 2.79) [p < 0.001]^†^	0.78 (0.71, 0.85) [p < 0.001]^†^	1.53 (1.26, 1.85) [p < 0.001]^†^	-0.21 (-1.23, 0.81) [p = 0.688]	1.90 (1.41, 2.55) [p < 0.001]^†^	0.62 (0.55, 0.69) [p < 0.001]^†^	2.20 (1.76, 2.76) [p < 0.001]^†^	0.39 (-1.05, 1.83) [p = 0.595]	3.35 (1.93, 5.82) [p < 0.001^†^]
African American/ White	1.79 (1.66, 1.92) [p < 0.001]^†^	1.62 (1.39, 1.90) [p < 0.001]^†^	0.60 (-0.32, 1.51) [p = 0.201]	0.76 (0.59, 0.99) [p = 0.038]^†^	1.76 (1.60, 1.94) [p < 0.001]^†^	1.64 (1.33, 2.01) [p < 0.001]^†^	0.87 (-0.20, 1.95) [p = 0.112]	0.73 (0.55, 0.97) [p = 0.032]^†^	1.83 (1.63, 2.05) [p < 0.001]^†^	1.65 (1.27, 2.14) [p < 0.001]^†^	-0.06 (-1.87, 1.75) [p = 0.946]	0.98 (0.51, 1.87) [p = 0.940]
Hispanic/ Non- Hispanic	2.86 (​​2.42, 3.38) [p < 0.001]^†^	1.31 (0.92, 1.87) [p = 0.136]	-2.46 (-4.56, -0.35) [p = 0.022]^†^	1.27 (0.67, 2.42) [p = 0.462]	2.21 (1.68, 2.90) [p < 0.001]^†^	0.79 (0.44, 1.41) [p = 0.423]	-4.82 (-7.98, -1.66) [p = 0.003]^†^	0.93 (0.40, 2.14) [p = 0.868]	3.41 (2.75, 4.23) [p < 0.001]^†^	1.83 (1.16, 2.89) [p = 0.010]^†^	0.06 (-2.76, 2.88) [p = 0.967]	3.69 (1.18, 11.50) [p = 0.024]^†^
Missing/ Non- Hispanic	1.63 (1.44, 1.85) [p < 0.001]^†^	1.51 (1.13, 2.02) [p = 0.006]^†^	-1.70 (-3.39, -0.02) [p = 0.047]^†^	1.11 (0.68, 1.82) [p = 0.669]	1.50 (1.24, 1.81) [p < 0.001]^†^	1.34 (0.87, 2.06) [p = 0.184]	-1.76 (-3.96, 0.43) [p = 0.116]	0.90 (0.51, 1.61) [p = 0.735]	1.75 (1.48, 2.08) [p < 0.001]^†^	1.70 (1.13, 2.55) [p = 0.011]^†^	-0.97 (-3.62, 1.69) [0.476]	2.23 (0.83, 6.04) [p = 0.113]

^†^significant at *α* = 0.05

*Hosp. = COVID-19 Hospitalization

Among diabetes patients, the odds of COVID-19 infection (OR = 0.91, 95% CI = [0.85, 0.96]), hospitalization (OR = 0.73, 95% CI = [0.64, 0.83]), and in-hospital mortality (OR = 0.75, 95% CI = [0.60, 0.93]) for females are lower than males. The odds for COVID-19 in-hospital mortality of male diabetes patients with complications is also significantly higher than female patients (OR = 0.73, 95% CI = [0.57, 0.93]). The odds of COVID-19 infection for senior diabetes patients is lower than adult patients (ORs = 0.64–0.70, 95% CI = [0.55, 0.73]-[0.66, 0.75]). However, if these patients are infected with COVID-19, their odds of hospitalization are approximately twice that of adult patients. These results about senior and adult patients are still observed within the diabetes group with and with no complications. If senior (65 and older) diabetes patients are hospitalized, their average length of stay is 1.79 days longer than younger adult (18–39 years old) patients. Not surprisingly, senior diabetes patients have significantly higher odds of COVID-19 in-hospital mortality than younger (OR = 4.26, 95% CI = [1.79, 10.12]) and older adult (40–64 years old) patients (OR = 2.15, 95% ci = [1.66, 2.79]). In particular, the odds for COVID-19 in-hospital mortality of senior patients is significantly higher than younger adults patients within the diabetes group with no complications (OR = 15.01, 95% CI = [1.89, 119.21]).

African American diabetes patients have higher odds of COVID-19 infection (OR = 1.79, 95% CI = [1.66, 1.92]) and hospitalization (OR = 1.62, 95% CI = [1.39, 1.90]) than White diabetes patients. This significant higher odds for infection and hospitalization of African American patients pertains within the diabetes group with and with no complications. However, if these patients are hospitalized, their odds of in-hospital mortality is lower than White patients (OR = 0.76, 95% CI = [0.59, 0.99]). The statistical significance of this ratio persists in those with diabetic complications. Hispanic diabetes patients have higher odds of COVID-19 infection than non-Hispanic patients (OR = 2.86, 95% CI = [2.42, 3.38]). However, the mean length of stay for Hispanic patients is 2.46 (95% CI = [-4.56, -0.35]) days shorter than non-Hispanic patients, respectively. Specifically, this mean difference of COVID-19 hospital length of stay is significantly shorter in Hispanic than non-Hispanic patients within diabetes group with complications (OR = -4.82, 95% CI = [-7.98, -1.66]). Furthermore, the odds ratios for COVID-19 hospitalization (OR = 3.41, 95% CI = [2.75, 4.23]) and in-hospital mortality (OR = 3.69, 95% CI = [1.18, 11.50]) between Hispanic and non-hispanic patients with no diabetes complications are statistically significant.

The significance of the results in Table 4 can be affected by small sample sizes in the diabetes patient population with COVID-19 hospitalization. Therefore, we provide the sample sizes for these patients in the considered demographics groups in Table 5.

**Table 5:** Sample sizes of diabetes patients with COVID-19 hospitalization across different demographic groups.

	All Diabetes Patients	Diabetes Patients with Complication	Diabetes Patients with No Complication
Female/Male	1228 / 1239	860 / 865	368 / 374
Senior (65+) / Younger adult (18–39)	1381 / 115	1090 / 53	291 / 62
Senior (65+) / Older adult (40–64)	1381 / 971	1090 / 582	291 / 389
African American/ White	1558 / 517	1137 / 391	421 / 126
Asian/ White	21 / 517	9 / 391	12 / 126
Hispanic/ Non- Hispanic	146 / 2114	58 / 1156	88 / 558
Missing/ Non- Hispanic	205 / 2114	110 / 1556	95 / 558

## Discussion

We first discuss our results regarding the association between diabetic complications and COVID-19 outcomes. This discussion is arranged by specific complication types. We then discuss demographic disparities among diabetes patients with complications and also among those without complications. In this discussion, diabetes patients with one or more complications are considered as a single group.

### The association between diabetic complications and COVID-19 outcomes

We observe that diabetes patients have higher odds for COVID-19 infection compared to non-diabetes patients. As diabetes patients are at higher risk for severe COVID-19 outcomes [43–45], a possible explanation for this observation is that diabetes patients are more likely to seek care when they experience COVID-19-related symptoms. Furthermore, studies have shown that infectious diseases, respiratory tract infections in particular, occur more frequently and severely in diabetes patients [46–48]. In addition to studying the overall diabetes patient population, we have also analyzed subpopulations with specific complications. Our results show that among all diabetes patients, those with renal complications have the highest odds for COVID-19 infection compared to non-diabetes patients. Health systems should dedicate more resources to prevent the infection of diabetes patients with such complications in order to help alleviate the medical surge caused by an infectious disease outbreak.

Among patients infected with COVID-19, we find that diabetes patients have a higher risk of hospitalization compared to non-diabetes patients. Diabetes is one of the most common underlying medical conditions in hospitalized patients with COVID-19 [5, 49]. Furthermore, we observe diabetes patients with COVID-19 hospitalization are at higher risk of in-hospital mortality. This result is in line with several other studies that suggest 30 to 40 percent of all COVID-19 deaths have occurred among people with diabetes [50–52]. We observe that among all diabetes patients, those with metabolic complications have the highest risks of COVID-19 hospitalization and in-hospital mortality compared to non-diabetes patients. In aDCSI, only severe conditions, such as diabetes mellitus with ketoacidosis, diabetes mellitus with coma, and diabetes mellitus with hypoglycemic coma, are classified as metabolic complications. Therefore, regardless of their COVID-19 status, such patients have higher risk for hospitalization and mortality. In addition, studies have shown that COVID-19 infection increases the risk of diabetic ketoacidosis [24, 25, 53]. This indicates a bidirectional relationship between diabetes with metabolic complications and increased risk for COVID-19 hospitalization and mortality. Hence, diabetes patients with metabolic complications should be prioritized for intervention when diagnosed with COVID-19.

In general, diabetes patients have a longer COVID-19 hospital length of stay than non-diabetes patients. Among diabetes patients with at least one of the seven complications considered in this study, only those with cardiovascular or peripheral vascular complications had a significantly longer hospital length of stay than non-diabetes patients. One possible explanation for this result is that these patients require more diagnostic tests than non-diabetes patients. This result can provide hospital administrators information on how to schedule their staff based on their current diabetic patient population. For instance, if a hospital system has historically observed more diabetic patients with cardiovascular or peripheral vascular complications during specific periods throughout the year, they could plan to staff more providers during those times.

### Demographic disparities among diabetes patients with complications

#### Male vs female

In Table 4, male patients have higher odds for COVID-19 infection, hospitalization and in-hospital mortality than female patients in the general diabetes population. Although male patients with diabetes complications still have higher odds for COVID-19 in-hospital mortality, there is no statistically significant difference in COVID-19 infection and hospitalization between male and female patients. In general, males have higher risk for COVID-19 infection [54, 55], which can explain the significant differences observed in COVID-19 infection and hospitalization between male and female diabetes patients with no complications. However, when they have diabetes complications, male patients may be less frequently engaging in behaviors that generate high-risk for contracting COVID-19. This can explain the insignificant difference in the odds for COVID-19 infection and hospitalization between male and female patients with diabetes complications. Finally, the course of illness due to COVID-19 infection is known to be more severe in males leading to higher risks for worse outcomes and death [56]. Diabetic complications can exacerbate these risks [10], explaining our observation that male patients with diabetes complications have higher odds for COVID-19 in-hospital mortality than females. This result demonstrates the effect of diabetes complications on demographic disparities can vary with respect to COVID-19 outcome. More specifically, demographic disparities in one COVID-19 outcome might decrease while disparities in another outcome might increase at the same time in the presence of diabetic complications.

#### Senior vs younger adults

In Table 4, senior adults have longer length of stay as well as higher odds for COVID-19 infection, hospitalization and in-hospital mortality compared to younger adults in the general diabetes population. The odds of seniors for COVID-19 in-hospital mortality remains significantly higher than younger adults in the diabetes group with no complications. This difference, however, is statistically less significant in the diabetes group with complications. One explanation for this result might be the small number of younger adults with diabetes complications and COVID-19 hospitalization in our data set (i.e., [53]). Furthermore, diabetes complications, which are associated with worse COVID-19 outcomes [10], might be predominantly determining the mortality risk of those patients resulting in less age specific differences. The hospital length of stay difference is no longer statistically significant between seniors and younger adults in both diabetes groups with and without complications. This result might again be due to the fact that our data include a small number of COVID-19 hospitalizations of the diabetic younger adults with complications (i.e., [53]) and without complications (i.e., [62]).

#### African American vs white

We find that African American patients have higher odds for COVID-19 infection and hospitalization than White patients in the general diabetes population. However, whites have higher odds for COVID-19 in-hospital mortality than African Americans. The statistical significance of this difference persists in those with diabetic complications and disappears among the diabetic population with no complications. This significance change is potentially due to smaller sample size and lower mortality rate in the no complications group. Specifically, the total number of African American and white patients with no diabetes complications is approximately one third those with complications in our data set. Furthermore, the association between race and risk of COVID-19 mortality is inconsistent in the literature. Some studies considering patients in Georgia conclude that there is no difference in the risk of COVID-19 in-hospital mortality between African American and White patients [57, 58]. On the other hand, other studies found that African Americans have a higher risk of COVID-19 in-hospital mortality compared to White populations [59, 60] or vice versa [61, 62]. These diverse conclusions suggest that the racial disparities in COVID-19 mortality may manifest differently across hospital systems and patient characteristics.

The study population in Gupta et al [59] includes patients from a single hospital serving a majority Black population with high poverty rates, and the study population in Asch et al [60] includes Medicare beneficiaries across the U.S. Ogedegbe et al [61] and Buikema et al [62] do not focus on individuals with specific socio-economic or demographic characteristics, thus their study populations are more similar to ours. Ogedegbe et al [61] suggested that observing lower in-hospital COVID-19 mortality rates in African American patients despite well-established higher mortality rates of these patients in the general population could be related to out-of-hospital mortality because African American populations are more likely to be uninsured and underinsured than White populations and thus have poorer access to care. Buikema et al [62] suggested that the higher in-hospital COVID-19 mortality rates in African American patients underscores the need to promote earlier entry of racial/ethnic minorities into the healthcare system for COVID-19 preventive services and treatment.

#### Hispanic vs non-Hispanic

In Table 4, the mean COVID-19 hospital length of stay of Hispanic diabetes patients is shorter than non-Hispanic diabetes patients. This result is in line with an analysis conducted by the Agency for Healthcare Research and Quality (AHRQ) in 2020 [63]. Interestingly, this disparity is magnified in the population with diabetes complications, whereas it is not statistically significant in the diabetes population without complications. A possible explanation for this result is that the Hispanic population in the US is more likely to be under-insured or uninsured [64]. Therefore, Hispanic patients have more limited access to the hospital care compared to non-Hispanic patients when longer hospitalizations are needed when diabetes complications exist. Although Hispanic patients with diabetes complications have higher odds for COVID-19 infection, there are no differences in the odds for COVID-19 hospitalization and in-hospital mortality between Hispanic and non-Hispanic patients with diabetes complications. Thus, diabetes complications, which are associated with worse COVID-19 outcomes [10], might be predominantly determining the COVID-19 severity in those infected patients resulting in less ethnic differences. There are significant differences in the odds for COVID-19 hospitalization and in-hospital mortality between Hispanic and non-Hispanic patients with no diabetes complications. Since the diabetes population with no complications is more similar to the general population, this result is driven by underlying health disparities between Hispanic and non-Hispanic populations [65–67]. Note that these differences in the odds for COVID-19 hospitalization and in-hospital mortality between Hispanic and non-Hispanic patients are not observed in the general diabetes population in our data.

### Conclusion and limitations

This study contributes to the existing literature on understanding risk factors for COVID-19 related to underlying medical conditions and demographic characteristics. Specifically, we demonstrate that the presence of diabetes complications increases the risks of COVID-19 infection, hospitalization, and worse health outcomes with respect to in-hospital mortality and longer hospital length of stay. Furthermore, we show the presence of health disparities in COVID-19 outcomes across demographic groups in our diabetes population.

There are some limitations to our work that can be considered in future research. First, our data does not include a primary diagnosis code for each encounter. Thus, the primary reason for a COVID-19 infection or hospitalization encounter could be due to COVID-19 or other medical conditions which can obscure the definition of these two outcomes. Second, our results may not be generalizable to other patient populations since our dataset comes from a single hospital system. Future studies are needed in order to compare results from different hospital systems. Another limitation is that we did not account for time since diagnosis of diabetes in our analysis. We utilized a patients’ medical histories to identify diabetes diagnosis but were unable to include the time at which this diagnosis was first made. By collecting more data future research could investigate the impact of time since diagnosis of diabetes on different COVID-19 outcomes. Finally, we have applied logistic and linear regression to estimate the odds ratio and mean differences of COVID-19 outcomes among patients with different diabetic complications. Another interesting area of future research would be to measure the importance of these diabetic complications as independent variables in prediction models. For instance, future work could utilize binary indicators of diabetes complications as variables in a decision tree or random forest model. Then, mean impurity decrease or permutation feature importance could be used to evaluate the importance of diabetic complications for predicting COVID-19 outcomes. This analysis however should incorporate additional system and population level factors, e.g., ventilator availability and vaccine coverage.
